# What Can We Learn from Protein-Based Electron Transport Junctions?

**DOI:** 10.1021/acs.jpclett.1c02446

**Published:** 2021-12-02

**Authors:** David Cahen, Israel Pecht, Mordechai Sheves

**Affiliations:** Weizmann Institute of Science, Rehovot 7610001, Israel

To explain what drives us to study electron transport (ETp) through electrode/protein/electrode solid-state junctions (cf. Figure 1) we present some of the reasons, mostly in the form of the following questions:

1. **Scientific curiosity:***How can electron transport take place through nonconjugated, flexible, polyelectrolytic macromolecules*? Answering this question is also driven by intense current interest to understand ETp via so-called bacterial nanowires.^1−3^
2. **Biological implications and relevance:***Can we learn from understanding ETp via proteins also about their role in biological electron transfer (ET)?*
3. **Physico-chemical insights***: Which constituting elements and properties of proteins are involved in effective electron transport?* The following can be singled out:
  a. primary, secondary, and tertiary structure;
  b. π-electron content and H-bonding character of amino-acid residues;
  c. cofactors and their redox properties; alternatively, these can be described in terms of:
    i. the (electronic) energy levels of a cofactor’s HOMO and LUMO;
    ii. the energy difference between these levels, and between each of these levels and the electrode Fermi level;^51^
    iii. the difference between the electrochemical potentials of the electrodes (= Fermi level) and of the protein (≈ redox potential^51^).
4. **Potential applications**: *Can proteins serve as components of electronic devices as part of true bioelectronics?*

**Figure 1:**
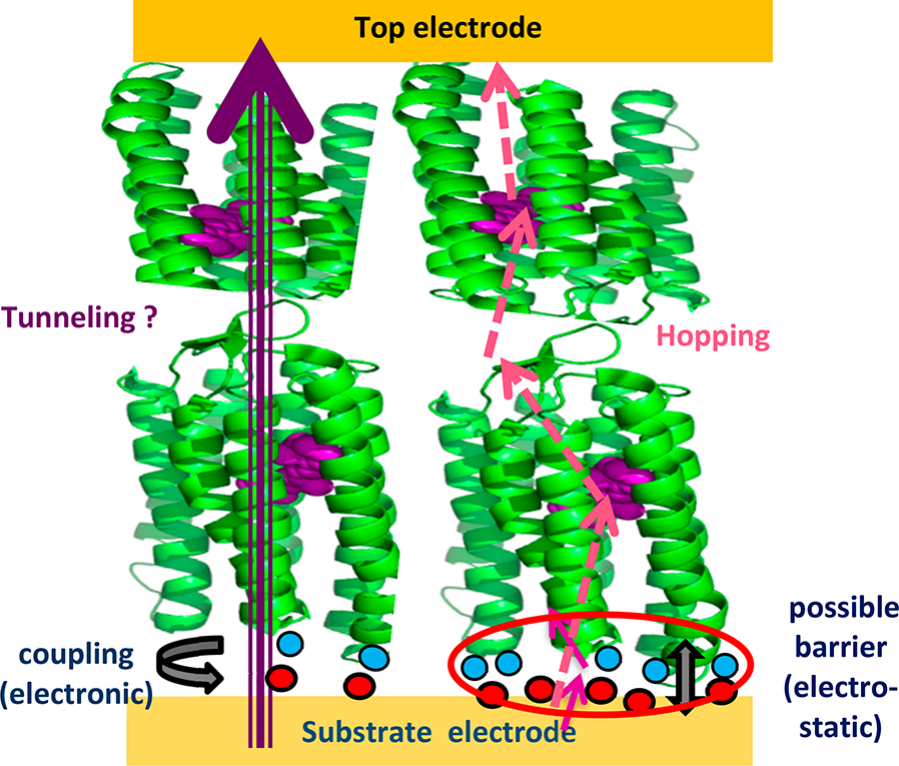
Illustrative scheme of a protein junction used for electron transport (ETp) measurements. (Adapted from ref (29). Copyright 2018 American Chemical Society). Cases with a low (electrostatic) barrier (left) and a strong one (right) are illustrated for a bilayer of the purple membrane proton-pumping protein, *bacteriorhodopsin* (bR; approximate length, 9 nm, i.e., well beyond expected tunneling distance; bR structure is from the protein data bank, PDB: 1fbb).

These questions led to an increasing number of studies, resulting in evidence for the relevance of the above motivations.^4−8^ At the same time, the results raise new, or leave open, existing issues. Herein, we discuss some of these results that we view as central and issues arising from questions 1–3 (question 4 is left for another occasion), with further questions presented in *italics*.

## 1 Tunneling as Transport Mechanism?

The central experimental observable is that ETp via several proteins is temperature (*T*)-independent,^9^ which is consistent with tunneling being the operating mechanism. Tunneling is here not just *a* mechanism, because for ET (see sections 4 and 5 for ET vs ETp) it will manifest a *Quantum effect* in biology.^10−12^ We can make an even stronger case for ETp by tunneling through the small proteins that were studied already for their ET, based on further experimental evidence from our studies (see section 3). However, *the observed T-independent ETp via larger proteins challenges our understanding*.

Tunneling implies a < femtosecond residence time of electrons^13^ near any nucleus, which is consistent with currents flowing without any change in atomic positions in the conducting protein. It is therefore an attractive mechanism for electron transport (over short distances). Tunneling is often invoked as the mechanism for ET over a ∼ 2 nm distance. Comparison of the ET and ETp time scales can be made (see first paragraph of section 4), using results obtained for the bacterial, photosystem I or II (PSI or PSII) reaction centers (RCs). There, the ≤2 ps time window of the first ET(p) step more than suffices for an electron to tunnel to the next energy minimum.^14^ This window also allows for possible longer characteristic times of (tunneling) transport via the complex medium that proteins present to electrons, a medium often viewed as electronically insulating.

Tunneling may be operating in *intra*molecular ET and ETp via proteins, over distances < ∼2.5 nm, which is also an established range for electron tunneling across/in insulators. In section 3, other results will be noted in support of ETp by tunneling over <2.5–3 nm wide protein junctions (similar to ET over < ∼2.5 nm^15,16^). *However*, *single step tunneling cannot rationalize experimental results of ETp across proteins, separating the electrodes by ≥ ∼5 nm.*

This conclusion is illustrated by the observation that ETp across conjugated organic molecules changes from *T*-independent tunneling below to thermally activated hopping above a separation of ∼4.5 nm.^17,18^ Actually, tunneling over longer distances was observed only in high-quality semiconductors (≤ ∼20 nm for III–Vs) or metals because of the larger extension of their (quasi)free electron wave function.

**Figure 2:**
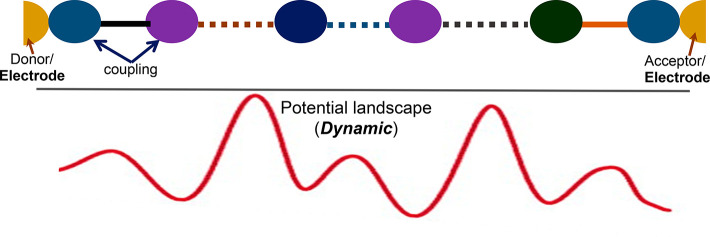
(Top) Scheme of a protein junction, depicted as arrangement of amino-acids and cofactors between electrodes (ETp) or between donor and acceptor (ET). Snapshot (<psec) of the electrostatic potential (bottom), which is dynamic and will change on a picosecond time scale. The average slope reflects the applied electrical potential. (Adapted from ref (4) and used with permission from the Institute of Physics.)

Another issue is that *quantum tunneling is a coherent process*, and it is not clear how to retain coherence for ETp across a dynamic as well as static disordered medium, such as proteins (cf. bottom part of Figure 2). Indeed, some recent models mostly do not deal with coherence^19−24^ or do not require it.^25^

Remarkably, though, the observed *T*-independence of ETp via proteins over ≥ ∼5 nm is consistent with results of optically monitored ET in frozen (glassy) protein solutions,^26^ and in single protein crystals,^27^ raising the question *what can be the mechanism of ETp over these longer distances, if not tunneling?* Several ideas addressing this question have been presented for organic molecules^19^ and proteins,^25^ but *how these are applicable to ETp via large proteins* is not clear. Still, as an electron current can also be viewed as a flow of holes in the opposite direction, aromatic amino acids, e.g., tryptophan and tyrosine,^28^ may play an important role because of their potential of hole formation and currents, as exemplified by hole hopping.

Thus, as further shown in section 3, tunneling explains ETp via small proteins, but for larger ones, understanding ETp still poses a challenge for theory, especially if we strive for models with predictive power.

## 2 Protein/Electrode Coupling at Their Interface (cf. Figure 1)

A troubling finding was that *T*-dependent protein ETp has been observed in some cases.^4^ This observation was explained by the role of protein–electrode coupling.^29^ The extent of current flow through a protein junction can be correlated with the height of a transport barrier the electrons encounter, if tunneling through the barrier is less probable than going over it. In the case we studied,^29^ an electrostatic barrier region of a few nanometers can exist at the electrode–protein interface. This may, under certain conditions, cause hopping over that barrier to dominate the ETp. Hopping over a barrier requires energy input (*E*_ac__t_), which explains why *T*-dependent transport is observed across the whole junction.^52^

The presence of π-electron-rich moieties in the protein will affect protein–electrode coupling, because the higher its electron density, the smaller the energy difference between the protein’s HOMO or LUMO energy level and the electrode’s Fermi level, which can present an *E*_act_ for ETp.

In summary, if *E*_act_ is negligible or the barrier is so narrow and low that tunneling through it dominates hopping over it, then charge flow between electrode and protein will be *T*-independent. This conclusion implies that, if experiment shows that the ETp *across the junction is temperature-independent*, then this entails that *electrons can pass through an energy barrier, posed by the protein, rather than having to transit over such a barrier* (see also section 5).

This now brings us to the question:

## 3 Is There Further Evidence for ETp via Tunneling?

### 3a Inelastic Electron Tunneling Spectroscopy (IETS)

Current via thin protein junctions (∼2 nm, e.g., of cytochrome c or azurin) carried by inelastic electron tunneling was found to constitute up to ∼1% of the total measured current.^30,31^ Importantly, this implies that 99% of the ETp is by elastic tunneling, supporting that, like ET, tunneling is the ETp mechanism over such distances.^15,16,32−34^ This distance limitation may be due to lack of significant tunneling component in ETp through the wider junctions (namely, via larger proteins), to poor S/N, or to limited junction stability. Hence, observing *IETS signals in ETp via wider protein junctions* constitutes an important future challenge.

### 3b On–Off Resonance Switching

The observation that an *increase* in applied voltage across a junction yields a *decrease* in current and, upon further voltage increase, the current is steeply increasing, is called in electronics negative differential resistance (NDR), and it is best known for tunnel diodes.

Underlying NDR is quantum tunneling across a barrier as this phenomenon is associated with getting the junction in and out of energetic resonance: in order to observe NDR, the applied voltage aligns empty (full) semiconductor (in our case protein) energy levels with full (empty) electrode levels, making them equi-energetic (ON resonance) or misaligning them (OFF resonance). The fact that NDR has been observed in the conductance–voltage characteristics of junctions of small proteins (∼2 nm)^35^ further supports the operation of ETp by tunneling across these junctions.

### 3c ETp Temperature Independence down to 4 K

ETp via the small protein Azurin could be measured all the way down to 4 K with no change observed from room temperature currents.^36^ This sets a limit on any barrier that the electrons would have to hop over, at ∼2–3 × *kT*, i.e., ∼1 meV. Interestingly, *T*-independence down to liquid He temperature fits with results of early ET measurements.^26^

In summary, even in the absence of (a way to determine) coherence, there is very strong evidence for tunneling as the dominant mechanism of ETp via smaller proteins. Being able to perform similar ETp measurements via larger proteins remains a challenge for the future.

## 4 Electron Transfer versus Electron Transport

Though ET and ETp seem and to some extent are similar (Figure 2, top part), there are important differences between them, which will be addressed here and in the last section. For a process where ET and ETp can be compared, we find (unpublished results) that ET rate values, *k*_ET_ (e^–^/sec, i.e., current), of the steps which are relevant for comparison are similar to the ETp currents: Measured ET rates in bacterial, PS I, and PS II RCs for the first steps after photoexcitation, before ion transfer (here proton coupling in the Q_A_ to Q_B_ step), were derived from optically measured processes. These values can be compared to those of the ETp, i.e., currents measured across PSI monolayers set between Au electrodes. The relevant ETp currents are those at an electrical potential difference, Δ*V*, where Δ*qV* (*q* = electron charge) is comparable to Δ(*E*_midpoint_)^37,53^ for ET. After approximate normalization (as e^–^/sec/protein), they are comparable, within an order of magnitude, to the sum of the ET rates measured for the above-mentioned steps. The sum of distances across these ET steps spans ∼6 nm, roughly comparable to that across which the ETp takes place (for time scales, see below).

In a broader review, we estimated (cf. ref (6)) the *k*_ET_ values that correspond to measured current densities across proteins at the low applied voltage of 100 mV and vice versa; we then plotted these versus ET distance (as used earlier in, for example, refs (15 and 16)). ET rates that correspond to the measured current densities can be estimated by assuming that a single ET channel is active in a cross section of 10 nm^2^, given protein cross section dimensions of ∼3 × 3 nm. The *k*_ET_/unit numbers, calculated from the macroscopic ETp data, are likely to be underestimated by 10^3^–10^4^, compared to STM^54^ (a bit less for conducting probe-AFM; nanowire contacts will be an intermediate case), because of the known differences between geometrical and actual contact areas of electrical contacts.^9^

### Redox Activity

ET involves resolvable chemical changes. ET into/from proteins involves redox-active cofactors, such as transition metal ions,^15,16^ organic redox centers (e.g., flavins^38^ and quinones), and electron-rich amino acids like cysteine, tyrosine, and tryptophan. In contrast, ETp via proteins does not involve redox activity, nor measurable chemical changes; it can occur also without cofactors, although with reduced efficiency.^39^ As illustrated by our study of the tetraheme STC protein, ETp involves this protein’s valence band levels, irrespective of whether they are involved in redox activity or not. What matters in ET is the latter type of levels, which will be confined to the very edges of the valence and conduction band extrema. For STC we found that ETp needs the heme-based levels, but not the Fe-based ones (see also section 5c). Note that this ET versus ETp difference may well bear on how to describe best the electron transmission, through-bond or through-space.

#### Charge Balance in ET and ETp

In ET, electron uptake or donation is followed by sub-ps electronic polarization, by >ps nuclear rearrangement, and then by permanent charge rearrangement that is slower by several orders of magnitude (a similar mechanism has been proposed for retinal proteins, stabilizing the light-induced dipole, formed in the retinal polyene upon light absorption^40^). On the longer time scales, charge balance is maintained by rearrangement of protein-bound ions, or exposure to the liquid electrolyte. This is also the case in electrochemical experiments (e.g., refs (41−43)), be they nano-, micro-, or macroscopic,^44^ because in all of those, part of the protein is exposed to the electrolyte. The same holds for recent work, where the electrical potential, applied via a reference electrode in solution, was kept well below that needed for a possible redox process, which is unlikely in this case because nonredox proteins were studied.^45^

No charge balance issue arises in optically induced ET experiments.^15,16^ The same holds for ETp because of the available large electron reservoirs (the electrodes).

#### Electronic Polarization in Proteins

Electronic polarization of the protein is important for charge stabilization during electron flow. An in-flowing electronic charge will traverse the whole length of a protein during just a few low-frequency natural vibration periods (∼30 to ∼100 cm^–1^; 0.3 to 1 ps). An electrical potential difference of 1 V (which is, in energy, Δ*qV* ≈ 1 eV) suffices for inducing electronic polarization in a nonionic solid. Across a protein, even lower applied bias voltages, Δ*V* < |0.5| V may suffice, also without polarizable cofactors, but with polarizable amino-acid side-chains. Moreover, it may be possible that charged amino acid residues can stabilize the electronic charge. In addition, bound H_2_O molecules (e.g., ref (46)) can help screen electrical charge imbalance by electronic polarization of the H_2_O lone pair.

In summary, much of the large differences that are observed between ETp- and ET-derived electron flow rates can be traced to the lack of or need for redox activity (more discussion on ET vs ETp can be found in refs (4 and 6)). However, while what is crucial for ET is of no or minor importance for ETp, viz., a redox center, this difference may shed new light on its role, as discussed next.

## 5 Electron Entry into and Exit from Proteins

After considering the latter steps in ETp and ET (in sections 5a and 5b, respectively), a conjecture will be presented in section 5c for the (lack of) need for a redox center, which concerns directly the biological process.

### 5a In ETp

In the absence of a barrier at the electrode–protein contact, the ETp process can be viewed as direct tunneling through the medium between metal contacts. Thus, when ETp proceeds by tunneling, currents are observed at potentials far below those known to effect chemical change. Hence, no *E*_act_ for electron transport via the protein is observed. This experimental result is also one of the consequences of a Landauer model-based theory.^25^ Thus, when ETp proceeds by tunneling, any electron residence time near the nuclei (none for pure tunneling) is too short to allow forming a new chemical species. Importantly, the electrodes’ presence assures a time-independent availability of “source” and “drain electrons”.

### 5b In ET

Redox-active proteins usually contain one or more cofactors, which serve as electron mediators, accepting and donating electrons, by switching between distinct redox states. Multiple cofactors set with < ∼2 nm separation allow for long-range electron transfer (as is well-illustrated in the above-mentioned photosynthetic reaction centers, or, for example, by (Ni, Fe) in hydrogenases).

### 5c The (Ir)relevance of a Redox Site in ETp

While both ET and ETp are driven by a difference of the electron’s electrochemical potential, in ETp this is electrical without a chemical contribution, and in ET it is chemical (without electrical contribution), necessitating a redox site. Indeed, the redox-capacity of cytochrome c did not affect the ETp it carried, as was shown by removing the Fe of its heme/hemin. Still, the porphyrin ring, redox-inactive in this case, *was* found to be crucial for efficient ETp.^47^ Electronic structure calculations showed that this is also the case for the earlier-mentioned ETp via the tetraheme protein STC.^48^

In ET, electron uptake by the acceptor site of the protein decreases the protein’s free energy, determined by the driving force of the reaction. The reduced site of the protein can also be oxidized and transfer the electron to an external acceptor, after overcoming an overpotential/activation energy. As these two processes are not coordinated in time, the redox center serves to “park” the electron until reaction partners are ready for the electron transfer step. The acceptor can be a dedicated molecule or another protein, where the two proteins need to be in optimal relative position with respect to each other for efficient ET to occur. In the absence of a redox site the electron’s excess energy may trigger undesired changes directly or via its complete conversion to heat.

As the electrodes are always there as electron reservoirs, the above-mentioned source/drain function of the electrodes implies that in ETp there is no issue of the timing of electron delivery or uptake. Electrons crossing the protein are removed before nuclear rearrangement can occur, and any electronic polarization is temporary. Therefore, during ETp, the protein’s nuclear and electronic structures remain intact, as is illustrated by cases where this can be ascertained in a junction for proteins whose function depends on their photoactivity, such as bR^49^ and YtvA.^50^ Thus, for ETp there is no need for a redox site, only for accessible empty/filled energy levels in the electrodes as well as in the protein. While the latter requirement is shared with ET, we noted in section 4 that one of the differences between ETp and ET lies in the VB and CB levels that dominate the process of the electron crossing the protein.

Naturally, in both cases the protein’s structure and its chemical properties do matter. *In which ways they do so, beyond those considered here, is one of the open questions for future research*.

## Conclusions

Proteins represent macromolecules with defined structures which can accomplish an essentially unlimited diverse, complex, and yet specific set of functions. Unexpectedly, they have been found to enable remarkable electron transport efficiencies which might be an “unintended consequence” of other properties which evolved for performing different functions. Thus, understanding their electron transport mechanism poses a major challenge. While ETp via relatively small proteins (< ∼2.5 nm) was found to be temperature-independent, consistent with tunneling as the mechanism, transport via larger ones also shows such independence, presenting a challenge of resolving its mechanism. Tunneling as mechanism was further supported by inelastic electron tunneling spectroscopy studies and by the observation of negative differential resistance (NDR). Rationalizing electron transport via proteins which separate electrodes by ≥ ∼5 nm by single-step tunneling runs counter to established physics. *Therefore, one major future challenge should be developing new models for the ETp mechanisms via such larger proteins, models with predictive power that can be tested experimentally, and, preferably, might also be falsifiable.*
